# The Snapdragon LATE ELONGATED HYPOCOTYL Plays A Dual Role in Activating Floral Growth and Scent Emission

**DOI:** 10.3390/cells8080920

**Published:** 2019-08-17

**Authors:** Marta I. Terry, Fernando Pérez-Sanz, Pedro J. Navarro, Julia Weiss, Marcos Egea-Cortines

**Affiliations:** 1 Genética Molecular, Instituto de Biotecnología Vegetal, Edificio I+D+I, Plaza del Hospital s/n, Universidad Politécnica de Cartagena, 30202 Cartagena, Spain; 2 Biomedical Informatic and Bioinformatic Platform, Biomedical Research Institute of Murcia, University Clinical Hospital ‘Virgen de la Arrixaca’, University of Murcia, 30120 Murcia, Spain; 3 Escuela Técnica Superior de Ingeniería de Telecomunicación (DSIE), Campus Muralla del Mar, s/n, Universidad Politécnica de Cartagena, 30202 Cartagena, Spain

**Keywords:** antirrhinum, aroma, artificial vision, biological rhythm, flower development, growth rate, phenomics, RNA interference, volatile organic compounds

## Abstract

The plant circadian clock controls a large number of internal processes, including growth and metabolism. Scent emission displays a circadian pattern in many species such as the snapdragon. Here we show that knocking down *LATE ELONGATED HYPOCOTYL* in *Antirrhinum majus* affects growth and scent emission. In order to gain an understanding of the growth kinetics, we took a phenomic approach using in-house artificial vision systems, obtaining time-lapse videos. Wild type flowers showed a higher growth speed than knockdown plants. The maximal growth rate was decreased by 22% in plants with lower *LHY* expression. Floral volatiles were differentially affected as RNAi plants showed advanced emission of compounds synthesized from cinnamic acid and delayed emission of metabolites of benzoic acid. The monoterpenes myrcene and ocimene were delayed, whereas the sesquiterpene farnesene was advanced. Overall, transgenic lines showed an altered volatile emission pattern and displayed a modified scent profile. Our results show that *AmLHY* plays an important role in the quantitative and qualitative control of floral growth and scent emission.

## 1. Introduction

Floral organs are arranged in whorls of sepals, petals, stamens, and carpels. They acquire their identities when a set of genes called floral organ identity genes are activated. These organ identity genes activate the morphogenetic program in combinations [1]. The MADS-BOX genes DEFICIENS and GLOBOSA form protein complexes that drive petal and stamen development [2,3]. The so-called C-function genes are involved in the organ identity of the stamens and carpels [4] and cause the termination of the floral meristem development [5,6]. 

Floral organ size is primarily controlled by the organ identity genes in a quantitative way [7,8]. Changes in floral organ size occur by changes in the time and/or rate of cell division and expansion [9,10]. Lateral organs grow by a combination of cell division and expansion. Several genes controlling these processes also play a role in the final size of the flower. Amongst them are the transcription factor *AINTEGUMENTA* [1,2,3,4], affecting both cell division and expansion and *EXPANSINS*, which control primarily cell expansion [11,12]. 

The emission of floral scent occurs in many cases from petal tissues [13] and has several functions including the attraction and guidance of pollinators and the repulsion of non-beneficial insects [14,15]. Apart of the important role of Volatile Organic Compounds (VOCs) emission in plant reproduction and protection, the general floral bouquet, which is perceived by the human olfactory system, is also of aesthetic value [16,17]. Floral volatiles are emitted in a circadian fashion and this fact characterizes some plants as day emitters, like *Antirrhinum* or roses, or night emitters, like *Petunia* or *Silene* [18,19,20], indicating that the majority of the volatiles are released during a given timeframe.

The circadian clock has been extensively studied in Arabidopsis [21]. Research on circadian clock in other species is increasingly contributing to the understanding of the genetics of the circadian clock in plants and the importance of its restructuring for optimal outputs in adaptation to the environment [22,23,24]. Cell expansion is controlled by the circadian clock through the coordination of DELLA (aspartic acid-glutamic acid-leucine-leucine-alanine) proteins and the PHYTOCHROME INTERACTING FACTOR (PIF) transcription factors [25]. Cell division is also under the direct control of the clock via binding of TIMING OF CAB 1 to the promoter of *CDC6* in Arabidopsis [26]. Scent emission is also directly controlled by the clock. Knocking down the *LATE ELONGATED HYPOCOTYL* (*LHY*) in *Petunia* causes the down regulation of the benzenoid/phenylpropanoid pathway [27], limiting the timing of floral volatile emission to the evening. Additional evidence for a direct role of the circadian clock on the regulation of floral scent emission has been found in *Nicotiana attenuate*, where knockdown of *LHY* or *ZEITLUPE* cause changes in the emission of the attractant benzyl acetone [28]. Recent work has shown that the ortholog of *ZEITLUPE* in *Petunia*, *PhCHANEL*, coordinates scent profiles [29]. 

In this work, we have addressed the function of the core clock gene *LHY* on flower development in *Antirrhinum majus* using RNA interference. Using a phenomics approach we analyzed the effect of knocking down *AmLHY* on growth. We also analyzed in detail the function of *AmLHY* on scent emission. Our results indicate a non-linear and complex control of the floral scent synthesis and emission process by *AmLHY*.

## 2. Materials and Methods

### 2.1. Phylogenetic Analysis

We obtained the *AmLHY* gene by BLAST (Basic Local Aligment Search Tool) against a set of genomic scaffolds using the Arabidopsis *LHY* as a query. We used the close relative gene from *Mimulus gutattus* (Migut.N01518.2.p) downloaded from Phytozome to obtain a cDNA model using Genewise [30]. The EMBL-EBI accession number was PRJEB24602.

LHY/CCA1 protein sequences were obtained from National for Biotechnology Information (NCBI) using BLASTp (Table S1). REVEILLE (RVE) Arabidopsis protein sequences were used as a query to identify these proteins in *Antirrhinum majus*, using the program tblastn and the genome version 3 in the Snapdragon Genome Database (http://bioinfo.sibs.ac.cn/Am/index.php), that had been recently released [31]. Protein alignment was performed by CLUSTAL X, a Windows interface for the ClustalW multiple sequence alignment software [32]. Phylogenetic trees were built with the R libraries “ape” and “phangorn” [33,34], Maximum Likelihood was used as a statistical method and Jones, Taylor, and Thornton (JTT) as a model of amino acids substitution [35] with 1000 bootstrap replicates. Trees were visualized with the R package “ggtree” [36]. Protein domains and functional sites were inferred using the web-based tool “PROSITE” (https://prosite.expasy.org/) [37] and the schematic structure of proteins was plotted with the Bioconductor package “drawProteins” [38]. All libraries were built under R version 3.5.1.

### 2.2. Plant Material and Transformation

We used the pHELLSGATE12 plasmid [39] to obtain a construct encompassing 156 bp, starting 45 bp before the predicted ATG. *Antirrhinum majus* hypocotyls of the Sippe 50 (Max-Planck-Institut für Pflanzenzüchtungsforschung, Köln, Germany) line were transformed using the *Agrobacterium tumefaciens* strain EHA105 as described previously [8]. Sippe 50 was an inbred line that had been used for genetic analysis since 1910 [40,41]. As it has been selfed since then it can be considered completely homozygous. Transgenic plants were confirmed by polymerase chain reaction (PCR) based on the amplification of the gene *NPTII*. Genomic DNA was isolated from leaves using a kit (NucleoSpin^®^ Plant II, Düren, Germany). PCR was conducted with OneTaq^®^ DNA Polymerase (see Table S3 for primers details). The PCR conditions were 30 s at 94 °C followed by 40 cycles of 15 s at 94 °C, 30 s at 60 °C, and 30 s at 72 °C and terminated by 5 min at 72 °C.

*A. majus* Sippe 50 and transgenic snapdragon seeds were germinated in vermiculite and were placed in a growth chamber with 16 h light/8 h dark cycle conditions at 23/18 °C during day and night, respectively. Transgenic plants were detected by PCR (NEB, One Taq DNA Polymerase, Ipswich, MA, USA) using *NPTII* primers (Table S3). We selected 3 independent lines: *RNAi:AmLHY14, RNAi:AmLHY26*, and *RNAi:AmLHY27*. Later, seedlings were transferred to pots with a mixture of peat, vermiculite, and perlite 1.1:1 in a greenhouse under natural conditions. Finally, seven days before sampling, plants were placed in a growth chamber with 12 h light/12 h dark cycles with 23/18 °C temperature cycles. Zeitgeber time 0 (ZT 0) was defined by the time when the lights turned on.

### 2.3. Gene Expression Analysis

Fully expanded leaves were collected from 3 to 4 plants at 6 h intervals for a complete day (24 h). Leaves were immediately frozen in liquid N₂ and stored at −80 °C until use. Total RNA from leaves was extracted using a phenol-based method as described in reference [42] and treated with DNase I (Fermentas-ThermoFisher, Waltham, MA, USA). Equal amounts of total RNA (ThermoFisher, NanoDrop 2000, Waltham, MA, USA) were used for cDNA synthesis (ThermoFisher, Maxima First Strand cDNA Synthesis Kit, Waltham, MA, USA). *AmLHY* expression levels were analyzed by quantitative polymerase chain reaction (qPCR) (Agilent Mx3000P QPCR System, Santa Clara, CA, USA) with SYBR Green (TaKaRa, SYBR Green Master Mix, Kusatsu, Shiga, Japan) according to manufacture manuals, and the following protocol was used for 40 cycles: 95 °C for 4 min and then cycling at 95 °C for 15 s, 60 °C for 15 s, and 72 °C for 30 s. *UBIQUITIN* (*AmUBQ*) was used as an internal control and samples were run in duplicate (Table S3). Normalized expression was expressed as 2^−ΔΔCt^ , thus we used an efficiency of 2 for data analysis [43]. Daily gene expression of *AmLHY* was normalized by the average expression level across the time-course; *AmLHY* expression in RNAi lines was normalized to WT. To test differences in expression between wild-type and transgenic plants we performed a Student’s *t*-test using the package “stats” in R (R version 3.5.1).

### 2.4. Flower Measurements

Flowers from *A. majus* Sippe 50 non-transgenic siblings and T2 generations were measured 2 to 3 days post-anthesis according to reference [44]. Flowers were sampled from 3 to 4 different plants per group. In total, we measured 8 flowers from non-transgenic siblings, 9 flowers from *RNAi:AmLHY14*, 4 flowers from *RNAi:AmLHY26*, and 7 flowers from *RNAi:AmLHY27* line. Floral parameters were compared between non-transgenic and transgenic siblings with the Student’s *t*-test performed in R (R version 3.5.1).

### 2.5. Growth Analysis and Artificial Vision

We used an image acquisition system described previously [45,46]. Plants were grown inside a growth chamber comprising LED day lights covering from UV to red light. Night images were taken by activating an infrared light at 840 nm wavelength during short intervals of time (3 s). Images were acquired with an artificial vision camera comprising two CCD sensors, a multichannel 24-bits RGB absorbing at 610 nm, 540 nm, and 460 nm, and a monochromatic sensor capturing at 800 nm. The acquired images have a resolution of 1296 × 966 pixels.

We obtained data using a light/dark 12 h/12 h period of *A. majus* Sippe 50 and the segregating siblings of *RNAi:AmLHY* for a period of 8 days and 7 h. Images were acquired every 10 min for a total of 1196 images comprising 3 wild type and 3 transgenic flowers. Using a semiautomatic process, we measured the length every two hours. A curve was fitted to the raw data using a GENERAL ADDITIVE MODEL (GAM) using the package “mgcv” from R. The maximum growth corresponded to the maximal size of the flowers, corresponding to the maximal value of the curve. We obtained the maximal slope that would correspond to the maximum growth rate by deriving 200 points of the curve and obtaining the highest number. The area under the curve was obtained by integrating the fitted curve using the integrative function from the “stats” package.

### 2.6. Volatiles Collection, Gas Chromatography Mass Spectrometry, and Scent Analysis

Non-transgenic plants and plants belonging to T2 generations were sampled. Three or four flowers per line from 2 to 3 days post anthesis were used as described previously [47]. We collected volatiles starting at ZT3, for a period of 6 h during 24 h. All plants were acclimated to 12 h light/12 h dark (12LD) and 18/23 ºC cycle. The volatiles captured were analyzed by gas chromatography mass spectrometry (GC/MS) as described in reference [8]. Flower scent was analyzed by the R package “gcProfileMakeR” (unpublished results), selecting a 60% frequency and default quality. Selected volatiles amount was expressed as an integrated area divided by flower fresh weight [48]. The phase was calculated using the JTK-CYCLE implemented in “MetaCycle” [49] (R version 3.5.1). Graphs were plotted with “ggplot2” [50] using a color blind-friendly palette provided by “viridis” [51]. Volatile pathways were represented using the software “PathVisio” (version 3.3.0) [52].

## 3. Results

### 3.1. The Antirrhinum Majus LHY

We obtained a single scaffold that had high similarity to the *LHY* gene from Arabidopsis. A multiple sequence alignment of the translated proteins showed a high level of homology of snapdragon LHY (AmLHY) to other LHY and REVEILLE (RVE) coding genes (Figure S1, Table S1). In total, we identified seven *RVEs* genes in snapdragon and one *LHY* by BLAST, with high similarity to Arabidopsis (Table S2). A phylogenetic reconstruction using predicted proteins showed that the AmLHY clusters with other LHY proteins and they were clearly separated from the RVE clade (Figure S1). Moreover, CCA1a and CCA1b from *Physcomitrella patens* clustered between the RVE1/2/7 and RVE4/8 clades. LHY/CCA1/RVE (AtLHY, AtCCA1, and AtRVE in Arabidopsis) are MYB proteins, which are characterized by a conserved DNA-binding domain [53,54,55] 

We identified the MYB domain and the HTH DNA-binding domain (helix-turn-helix motif) in AmLHY, which were highly conserved in size and position, as observed in other species such as *Petunia* or *Vigna* (Figure S1C). The coding region of AmLHY differed compared to AtLHY. AmLHY showed additional aminoacids that resulted in a longer protein (Figure S2). As mentioned above, we identified seven genes related to the RVE family in snapdragon, which shared a high homology with Arabidopsis except for three genes. We could not identify the MYB domain in AmRVE1/2/7A and AmRVE1/2/7B, while AmRVE1/2/7D showed two MYB domains (Figure S1C), suggesting an internal gene duplication.

### 3.2. The AmLHY Shows A Diurnal Expression Pattern

**Figure 1:**
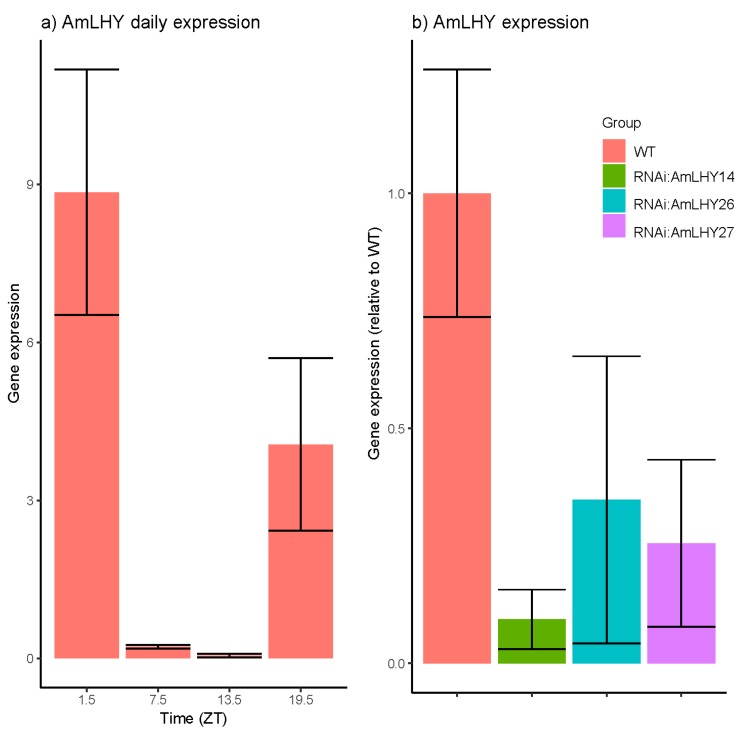
*AmLHY* expression: (**a**) Daily expression in wild-type plants under a 12 h light/12 h dark (12LD) cyle; (**b**) *AmLHY* expression at ZT1.5 in wild-type (red) and transgenic lines *RNAi:AmLHY14* (green), *RNAi:AmLHY26* (blue) and *RNAi:AmLHY27* (purple). Expression was determined by the ΔΔCt method using the wild-type as a control. Reference gene was *AmUBI*. Each bar represents the average ± standard deviation from 3 to 4 biological replicates.

We analyzed the expression of *AmLHY* in leaves of wild-type plants under a 12LD cycle. We found that *AmLHY* expression increased at the end of the subjective night, peaking in the early morning. *AmLHY* showed a robust and significant oscillation (Figure 1a, *p* value = 3.3 × 10^−06^), as described in other species including Arabidopsis, cowpea, or wild tobacco [54,56,57]. We selected T1 plants from three independent lines (*RNAi:AmLHY14*, *RNAi:AmLHY26* and *RNAi:AmLHY27*). In T2, all selected lines displayed a significant lower transcript level of *AmLHY* (Figure 1b, Table 1). *RNAi:AmLHY14* showed an average of 90% reduction in *AmLHY* expression levels, *RNAi:AmLHY26* of 65% and finally, *RNAi:AmLHY27* of 74%.

### 3.3. AmLHY Does Not Affect Flower Morphology and Size

The typical snapdragon flower has five sepals and five petals, these petals are fused resulting in a tubular and bilaterally symmetrical flower. We did not observe changes in organ identity or aberrant growth. All plants showed fully opened flowers.

We characterized snapdragon flowers analyzing 11 floral parameters, comparing the wild-type Sippe 50 and *RNAi:AmLHY* lines. These measurements included sepals, petals, and reproductive organs. The down regulation of *LHY* did not significantly affect the flower size, with the exception of petal height, lower petal expansion, and palate, which decreased in *RNAi:AmLHY14* line (Figure S3, Table S4).

### 3.4. AmLHY Enhances Growth Speed

**Figure 2:**
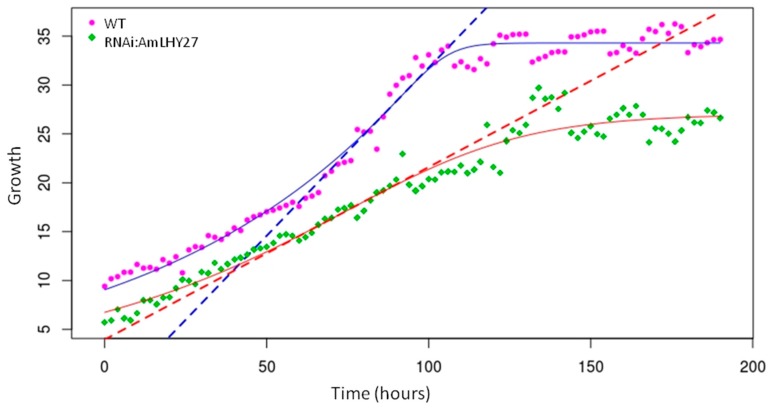
Growth curve, expressed in mm, of flowers of non-transgenic (WT, purple) and *RNAi.AmLHY27* (green). Dots represent the raw data, solid lines indicate the adjusted curve and dashed lines denote the maximum slope, that corresponds to the maximum growth rate.

The study of mutants affecting floral size in Arabidopsis, *Antirrhinum*, and other species have relied mostly on endpoint analysis of mutants [10,58]. There is very little information about the growth kinetics that causes the final changes in terms of size. Modifications in growth leading to differences in final size may occur as a result of the duration of the growth period, changes in growth speed, or a combination of both parameters. We used a previously described in-house artificial vision system to obtain time lapse pictures of line *RNAi:AmLHY27* and an azygote segregating sibling (Movie S1). We obtained growth curves for wildtype and transgenic siblings and results showed a very strong effect of the down regulation of *AmLHY* on flower growth rate (Figure 2). The maximal growth speed achieved by the *RNAi:AmLHY* line was roughly 78% of the non-transgenic sibling, and the area under the growth curve model was 73% of the non-transgenic sibling (Table 2). The maximal slope used to calculate growth rate [59] suggested that the overall growth rate was only 51% as compared to wild type. As far as we could analyze, the length of the flower growth period was similar in WT and in *RNAi:AmLHY27*, indicating that modifying the plant circadian clock affected growth speed in the petals.

### 3.5. AmLHY is Required for Major Volatile Production

We analyzed the floral scent emitted by wild-type and *RNAi:AmLHY* flowers using a time course with sampling six hours apart. A collection of volatiles was performed under a 12LD cycle. We detected 15 compounds during the time-course sampling (Table S5). Most detected volatiles belonged to the phenylpropanoid/benzenoid pathway but we also found terpenes as described previously [60]. We divided the volatile organic compounds into two groups. The first group comprised those VOCs, which contributed to the scent profile above 2% (major volatiles) and the second group contained the remaining compounds (minor volatiles).

The major group comprised five compounds: Acetophenone, methyl benzoate, 3,5-dimethoxytoluene, ocimene, and linalool. We found major changes in the overall profile (24 h) in *RNAi:AmLHY*. The monoterpenes ocimene and linalool increased their levels in transgenic lines by 34.5% and 46.16%, respectively. In contrast, the volatile 3,5-dimethoxytoluene contribution to the scent profile was reduced by 23.6% in RNAi plants. Acetophenone and methyl benzoate, the two major compounds, decreased but not significantly in RNAi lines (2.6 and 7.5% respectively) (Figure S4). These five compounds comprised 98.35% of the profile in wild-type plants and an average of 97.81% in *RNAi:AmLHY* lines. Interestingly, the minor volatiles, whose contribution to the fragrance aroma was 1.65% in WT and 2.19% in transgenic lines, revealed the highest complexity of the snapdragon aroma. We classified the volatiles into two groups. The first group comprised compounds which decreased in the profile: The aldehydes nonanal and decanal. The second group included the benzenoids/phenylpropanoids 2-hydroxyacetophenone, benzaldehyde, cinnamyl alcohol, ethyl benzoate, methyl cinnamate, and methyl salicylate, and the terpenes farnesene and myrcene, which increased their contribution to the odour (Figure S4).

**Figure 3:**
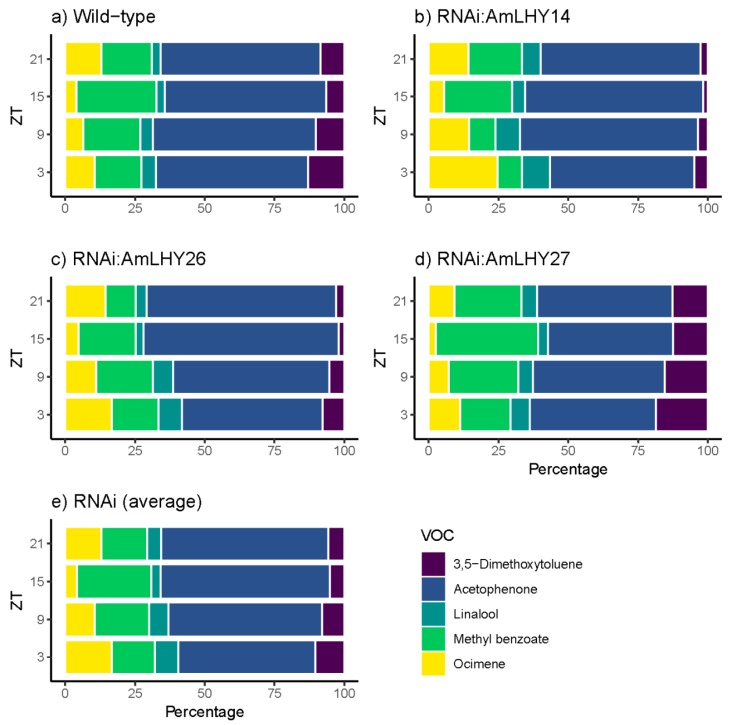
Daily scent emission of major compounds in wild type (**a**), and transgenic lines *RNAi:AmLHY14* (**b**), *RNAi:AmLHY26* (**c**), *RNAi:AmLHY27* (**d**). An average of RNAi lines is represented in (**e**). ZT indicates *zeitgeber* time.

Daily emission also revealed changes in the scent profile. In major compounds, we found that the contribution of the benzenoids acetophenone and 3,5-dimethoxytoluene to the scent profile was lower in RNAi lines along all the time points analyzed. Methyl benzoate level was lower in all time points, except at ZT15, which increased its contribution slightly. In contrast, the monoterpenes linalool and ocimene were higher in transgenic lines (Figure 3).

**Figure 4:**
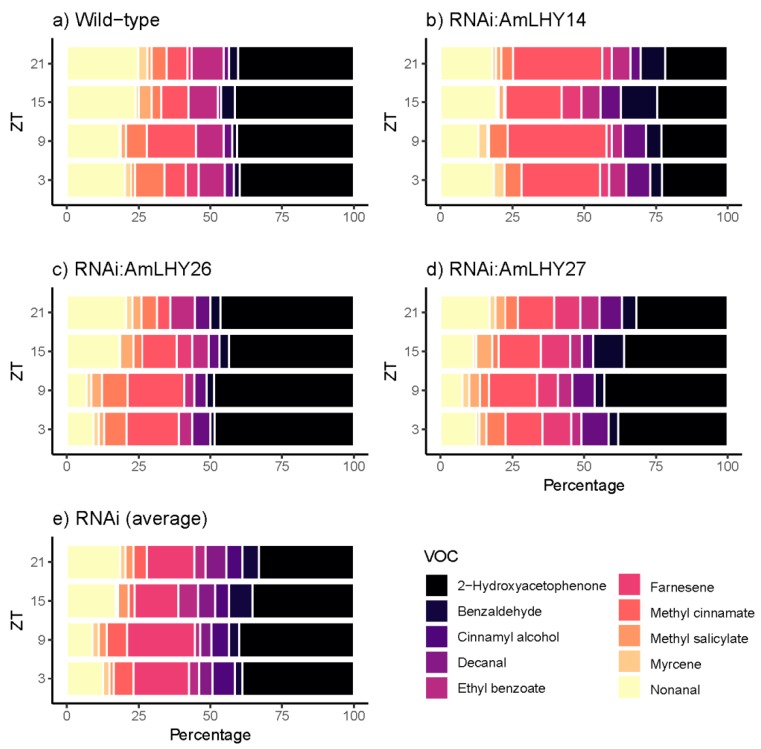
Daily scent emission of minor compounds in wild type (**a**), and transgenic lines *RNAi:AmLHY14* (**b**), *RNAi:AmLHY26* (**c**), *RNAi:AmLHY27* (**d**). An average of RNAi lines is represented in (**e**). ZT indicates *zeitgeber* time.

We also observed changes in minor volatiles. The aldehydes nonanal and decanal contribution were lower in transgenic lines. Furthermore, the benzenoid/phenylpropanoids benzadelhyde, cinnamyl alcohol, and methyl salicylate levels were higher in *RNAi:AmLHY* lines. The remaining compounds, 2-hydroxyacetophenone, ethyl benzoate, methyl cinnamate, and myrcene did not show a robust pattern as myrcene, that showed the highest increment by 668% at ZT9, but decreased by 33% at ZT15 and by 37% at ZT21 (Figure 4).

Altogether these results suggest a complex regulation of synthesis and/or emission of volatile organic compounds by *AmLHY*, acting at different levels over the phenylpropanoid/benzenoid and terpene pathways. All these changes resulted in a different and new flower-odour blend.

### 3.6. AmLHY Controls the Timing of Scent Emission

We detected 15 volatile organic compounds (Table S5; Figure 3 and Figure 4). In wild-type flowers, most selected volatiles showed a diurnal pattern. The release of VOCs started in the early morning, reaching their maximum emission at midday or before dusk. We classified the emitted VOCs in three groups based on their time of maximum emission. The first group comprises myrcene, ocimene, linalool, decanal, nonanal, 3,5-dimethoxytoluene, 2-hydroxiacetophenone, and methyl cinnamate, that reached their maximum emission at early day and midday. A second group included cinnamyl alcohol and farnesene that peaked at the end of the light period. Finally, the third group covered acetophenone, methyl benzoate, and methyl salicylate, which increased their emission at midnight (Table S6).

In contrast, we found that the maximum emission of some VOCs shifted in RNAi lines, and we divided the volatiles into three groups. First, the monoterpene linalool, the aldehydes nonanal and decanal and the benzenoids/phenylpropanoids cinnamyl alcohol, 3,5-dimethoxytoluene, and 2-hydroxyacetophenone did not present any differences with wild-type flowers, showing its maximum emission at the same time. The second group comprised the derivatives of benzoic acid methyl benzoate and methyl salicylate, and the monoterpenes myrcene and ocimene, which delayed their maximum emission. Finally, acetophenone, benzaldehyde, methyl cinnamate (except in line *RNAi:AmLHY26*, which did not show any difference) and the sesquiterpene farnesene peaked early in transgenic lines (Table S6). We could not determine the phase of the ethyl benzoate, due to their irregular emission (Table S6).

**Figure 5:**
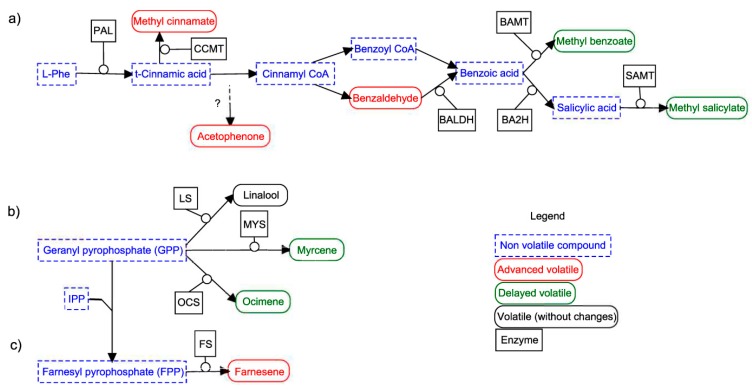
Schematic view of some phenylpropanoids/benzenoids (**a**), monoterpenes (**b**) and sesquiterpenes (**c**) pathway. Non-volatile compounds are represented in a dashed and blue rectangle and volatile compounds with a solid line and a rounded rectangle, the green color indicates a volatile that displays a delayed emission and the red color, an advanced emission. Enzymes and the C_5_ unit IPP (isopentyl diphosphate) are shown over the arrows. Enzyme abbreviations: BA2H (benzoic acid 2-hydroxylase), BALDH (benzaldehyde dehydrogenase), BAMT (benzoic acid carboxyl methyl transferase), CCMT (cinnamic acid carboxyl methyl transferase), FS (farnesene synthase), LS (linalool synthase), MYS (myrcene synthase), OCS (ocimene synthase), PAL (phenylalanine ammonia lyase) and SAMT (salicylic acid carboxyl methyl transferase).

Based on the known phenylpropanoid/benzenoid pathway we divided it into two principal branches, which have a common precursor, L-phenylalanine. The first branch included those compounds that derived from trans-cinnamic acid, as methyl cinnamate. The second branch covered benzoic acid derivatives, methyl salicylate, and methyl benzoate (Figure 5). Our results showed that the down-regulation of *AmLHY* advanced the maximum emission of acetophenone, benzaldehyde, and methyl cinnamate, while it caused a delayed peak of methyl salicylate and methyl benzoate.

We found a similar pattern in terpene emission. Geranyl pyrophosphate (GPP) is the precursor of the monoterpenes linalool, myrcene, and ocimene [61], whose reactions are catalyzed by linalool synthase (LS), myrcene synthase (MYS) and ocimene synthase (OCS), respectively. RNAi flowers displayed a delayed emission of myrcene and ocimene, peaking at midday (ZT6). In contrast, linalool was not affected. The emission of farnesene, a sesquiterpene that derives from farnesyl pyrophosphate (FPP) by addition of isopentyl diphosphate (IPP) to GPP [62], was advanced in transgenic snapdragons (Figure 5, Table S6).

Altogether, our results suggest that *AmLHY* regulates the synthesis and/or emission of volatile compounds at different levels. First, principal changes were found in minor compounds contribution, which comprises the highest diversity fraction of snapdragon aroma. Second, the emission of those VOCs derived from trans-cinnamic acid (benzaldehyde and methyl cinnamate) started earlier, whereas the compounds that derived from benzoic acid (methyl benzoate and methyl salicylate) started later. The terpenes myrcene and ocimene, which shared the precursor GPP, also displayed a delayed emission phenotype, while farnesene peaked early in RNAi lines.

## 4. Discussion

In this work, we have performed a functional analysis of the *Antirrhinum majus LHY* gene. The *LHY* gene is found as a single copy gene in many plants such as *Petunia*, Arabidopsis, tomato, or *Nicotiana attenuata* [23,57]. Our results indicate that in *Antirrhinum* there is a single gene that may have similar functions to the ones described in Arabidopsis, *Petunia*, or *Nicotiana*, including control of growth and scent emission. In this work, we have uncovered a function in floral development where it enhances growth speed.

We found that loss of function of *LHY* in *Antirrhinum* does not affect organ identity. Despite the importance of the plant circadian clock on the maintenance of the photosynthetic apparatus [63,64] , our results indicate that the plant circadian clock is not involved in the degradation of the chlorophyll machinery during petal development. Sepaloid petals result from decreased activity of B function genes and their targets [65], but we could not find evidence for an effect of *AmLHY* on petal identity. The transcriptional structure of the plant circadian clock varies between tissues and possible changes of the plant clock in petals versus other tissues or organs remains to be determined.

The size of plant lateral organs is the result of a combination of cell division and expansion. A variety of mutants affecting these processes in *Antirrhinum* show that petal growth can be differentially affected by genes indicating an intricate coordination of cell division, cell expansion, and compensation mechanisms [66]. The plant circadian clock is known to control cell expansion, our results demonstrate that *AmLHY* did not affect equally all the floral parameters analyzed. Our results did not differ from the findings in nightshades as down regulation of *LHY* does not affect floral size, petal junction distance, and corolla length, in *Nicotiana attenuata* or *Petunia* [27,28].

The temporal timing of cell division and expansion plays a key role in plant development. Both the length of the growth period and growth rate determine the final size of an organ. Our phenomic analysis of the *Antirrhinum* flower shows that the growth period is not controlled by *AmLHY* whereas the growth rate is substantially reduced. We may conclude that the period between cell divisions could be elongated in *RNAi*:*AmLHY* plants, resulting in organs with fewer and/or smaller cells. This hypothesis may require additional testing. Recent studies indicate that *LHY* represses abscisic acid (ABA) synthesis and signaling pathways [61], indicating that the newly identified effect of *AmLHY* on growth may occur via modulation of ABA. Our results are congruent due to the slow growth rate observed in RNAi plants highlighting the role of the circadian clock in flower development. The fact that floral organ size is not significantly reduced but growth speed is slowed in *AmLHY* knockdown lines indicates that compensation in terms of growth time may be occurring in order to achieve wild type organ size.

Floral scent profiles play a key role in the interaction of plants with both pollinators and pests [14]. The volatile blend is thought to be a complex interphase of interaction, and it is specific for a species [60]. The *A. majus* scent profile is highly complex as it comprises phenylpropanoids/benzenoids, and terpenes amongst the major VOCs. Previous studies showed that silencing *ZTL* in *Nicotiana atenuatta* causes a major reduction of benzyl acetone while *LHY* causes an early emission. The down regulation of *PhLHY* causes an earlier emission and reduction of methyl benzoate and benzyl benzoate [27,28]. Interestingly, bumblebees, one of the *A. majus* pollinators, are capable of detecting variations in aroma blends among snapdragon species, and wild *Antirrhinum* emitting different scent blends appear to have specialized pollinators [67,68]. As silencing *AmLHY* resulted in new aroma ratios, it may alter pollinators behaviour. Future studies may address the effect of silencing *AmLHY* and the interaction with pollinators as well as pests and/or pathogens.

We found that *AmLHY* plays a complex and central role in the coordination of floral scent emission, affecting timing and scent profile. While it does not have a major effect on the total emission, it has a profound effect on the emission of single volatiles. An important difference between *Antirrhinum* and the Solanaceae *Nicotiana* and *Petunia* is that it emits floral volatiles preferentially during the day. In contrast to previously published work in *Petunia* [27], we found that the down regulation of *AmLHY* caused a delayed emission of methyl benzoate (Figure 5). We can conclude that as the expression patterns of *AmLHY* and *PhLHY* are identical, the opposite effect on individual scent components emission timing is probably coordinated by a second layer of transcription factors. As the emission of single volatiles from the phenylpropanoids/benzenoids and monoterpene/sesquiterpenes is advanced or delayed in a consistent manner it appears that there might be either a difference at the transcriptional level or at the actual metabolic flux, giving rise to the observed differences. These two scenarios are not mutally exclusive and future research should help us understand the fine tuning of scent emission by *AmLHY*.

## 5. Conclusions

Our work shows that *AmLHY* plays a role in growth and scent emission. The combination of floral organ identity and circadian control are main factors required for proper flower development. Our results also revealed that the complexity of floral aroma depends on timing emission, which is under circadian clock regulation, and the contribution (or amount) of every single volatile. Interestingly, the down-regulation of *AmLHY* affected volatiles biosynthesis pathways at different branches. Timing of emission is an additional variable that may be well worth testing in plant-insect interactions.

## Figures and Tables

**Table 1:** *AmLHY* expression in wild-type and RNAi lines at ZT 1.5. Expression was normalized by WT. Average and standard deviation (SD) represents 3 to 4 biological replicates. The knock down percentage in RNAi lines is shown in the % KD column. Differences were tested by a Student’s t-test; a *p*-value <0.05 indicates a significant down-regulation.

Group	Average	SD	% KD	*p*-Value
WT	1	0.263		
RNAi:AmLHY14	0.093	0.063	90.66	0.001
RNAi:AmLHY26	0.348	0.306	65.23	0.006
RNAi:AmLHY27	0.256	0.178	74.44	0.003

**Table 2:** Parameters obtained from the phenomic analysis. Maximum growth corresponds to final maximum of the curve in mm. The maximum slope indicates the growth rate. The area under the model is an estimation of the difference in accumulated growth.

Group	Max. Growth/Std. Error	Max. Slope/Std. Error	Area
WT	34.301/0.191	0.344/0.021	4892.457
RNAi:AmLHY27	27.014/0.531	0.176/0.0078	3604.565

## Supplementary Materials

The following are available online at https://www.mdpi.com/2073-4409/8/8/920/s1, Movie S1: Snapdragon flowers growth, Figure S1: Phylogenetic reconstruction of AmLHY, LHY, and RVE predicted proteins, Figure S2: AtLHY and AmLHY protein alignment, Figure S3: Floral parameters in non-transgenic (WT) and transgenic lines (*RNAi:AmLHY*), Figure S4: 24 h scent profile, Figure S5: Total volatile amounts, Table S1: Accession numbers of proteins used in phylogenetic reconstruction, Table S2: BLAST analysis, Table S3: PCR primers used in the current study, Table S4: Analysis of floral parameters in non-transgenic and transgenic lines, Table S5: Retention time in minutes Table S6: Analysis of daily emitted volatiles with the algorithm JTK_CYCLE.
