# Supplementary material for: The Snapdragon LATE ELONGATED HYPOCOTYL Plays A Dual Role in Activating Floral Growth and Scent Emission
Source: Cells. 2019 Aug 17;8(8):920. doi: 10.3390/cells8080920 (PMC6721690; doi:10.3390/cells8080920)
Supplement: Supplementary file 1 [file cells-08-00920-s001.zip › SupplementaryCellsTerryetal/Supplementary Material Terry et al - AmLHY.pdf]

Supplementary files. Terry et al., “The snapdragon LATE ELONGATED HYPOCOTYL plays a dual role in activating floral growth and scent emission”

Supplementary Figure S1. Multiple sequence alignment of the predicted AmLHY and other proteins belonging to the LHY/CCA1 family. Vu (*Vigna unguiculata*), Vr (*Vigna radiata*), Pv (*Pisum vulgaris*), Gm (*Glycine max*), At (*Arabidopsis thaliana*), Na (*Nicotiana attenuata*), Ns (*Nicotiana sylvestris*), Ph (*Petunia hybrida*), Am (*Antirrhinum majus*), Amt (*Amborella trichopoda*), Zm (*Zea mays*), Os (*Oryza sativa*), Hv (*Hordeum vulgare*), Pp (*Physcomitrella patens*), Ot (*Ostreococcus tauri*).

Supplementary Figure S2. AtLHY and AmLHY protein alignment.

Supplementary Figure S3. Floral parameters in non-transgenic (WT) and transgenic lines (*RNAi:AmLHY*). Asterisk (*) denotes significant differences among wild type and transgenic flowers (see Supplementary Table S4).

Supplementary Figure S4. 24-h scent profile. Major (A) and minor compounds (B) of wild-type, RNAi lines and its average are represented in bars.

Supplementary Figure S5. Total volatile amounts, expressed as area divided by flower fresh weight, of wild type (WT) and transgenic lines (RNAi:AmLHY).

Figure S1

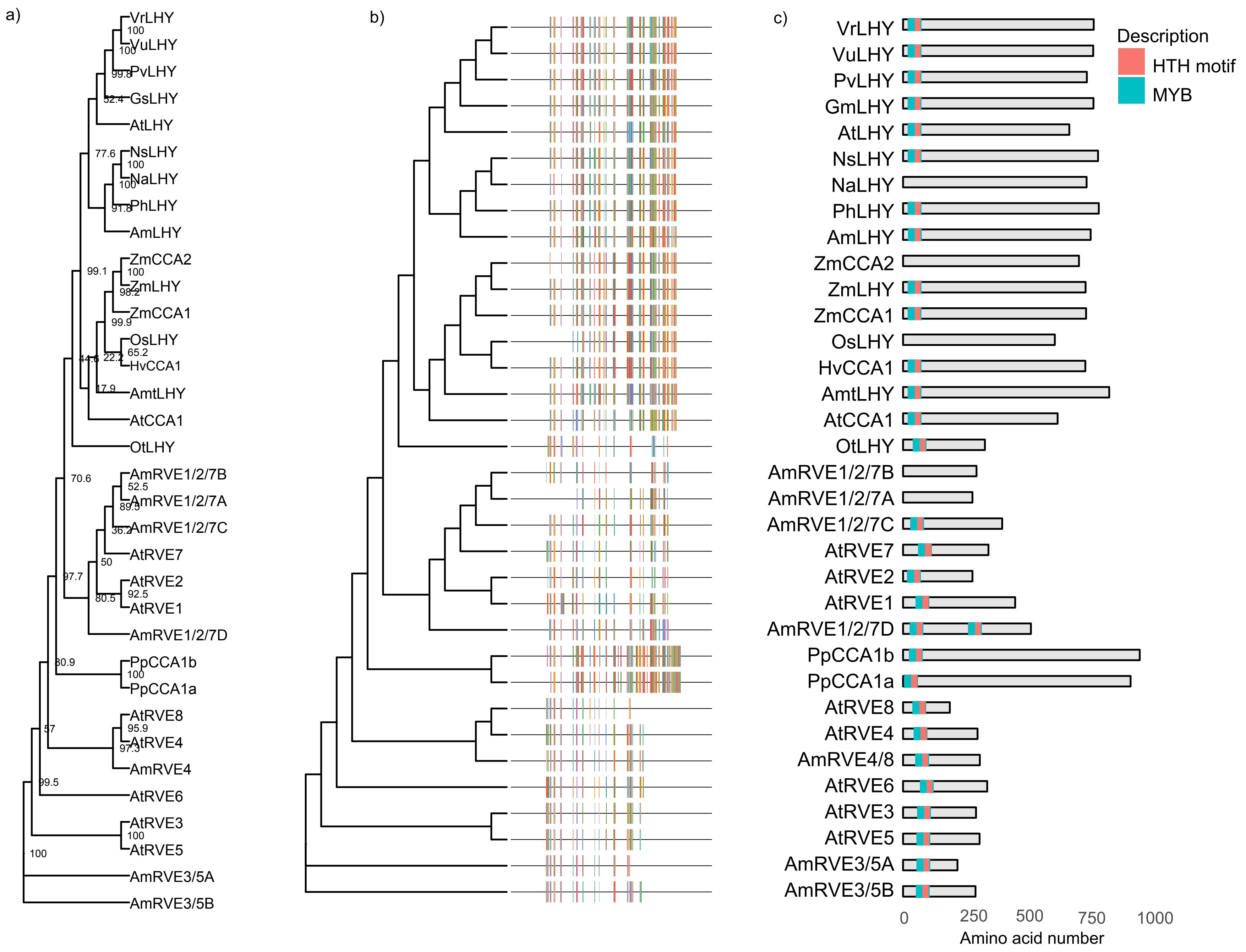
a)
VrLHY
VuLHY
PvLHY
GsLHY
AtLHY
NsLHY
NaLHY
PhLHY
AmLHY
ZmCCA2
ZmLHY
ZmCCA1
OsLHY
HvCCA1
AmtLHY
AtCCA1
OtLHY
AmRVE1/2/7B
AmRVE1/2/7A
AmRVE1/2/7C
AtRVE7
AtRVE2
AtRVE1
AmRVE1/2/7D
PpCCA1b
PpCCA1a
AtRVE8
AtRVE4
AmRVE4
AtRVE6
AtRVE3
AtRVE5
AmRVE3/5A
AmRVE3/5B
b)
c)
VrLHY
VuLHY
PvLHY
GmLHY
AtLHY
NsLHY
NaLHY
PhLHY
AmLHY
ZmCCA2
ZmLHY
ZmCCA1
OsLHY
HvCCA1
AmtLHY
AtCCA1
OtLHY
AmRVE1/2/7B
AmRVE1/2/7A
AmRVE1/2/7C
AtRVE7
AtRVE2
AtRVE1
AmRVE1/2/7D
PpCCA1b
PpCCA1a
AtRVE8
AtRVE4
AmRVE4/8
AtRVE6
AtRVE3
AtRVE5
AmRVE3/5A
AmRVE3/5B
Description
HTH motif
MYB
0
250
500
750
1000
Amino acid number

Figure S2

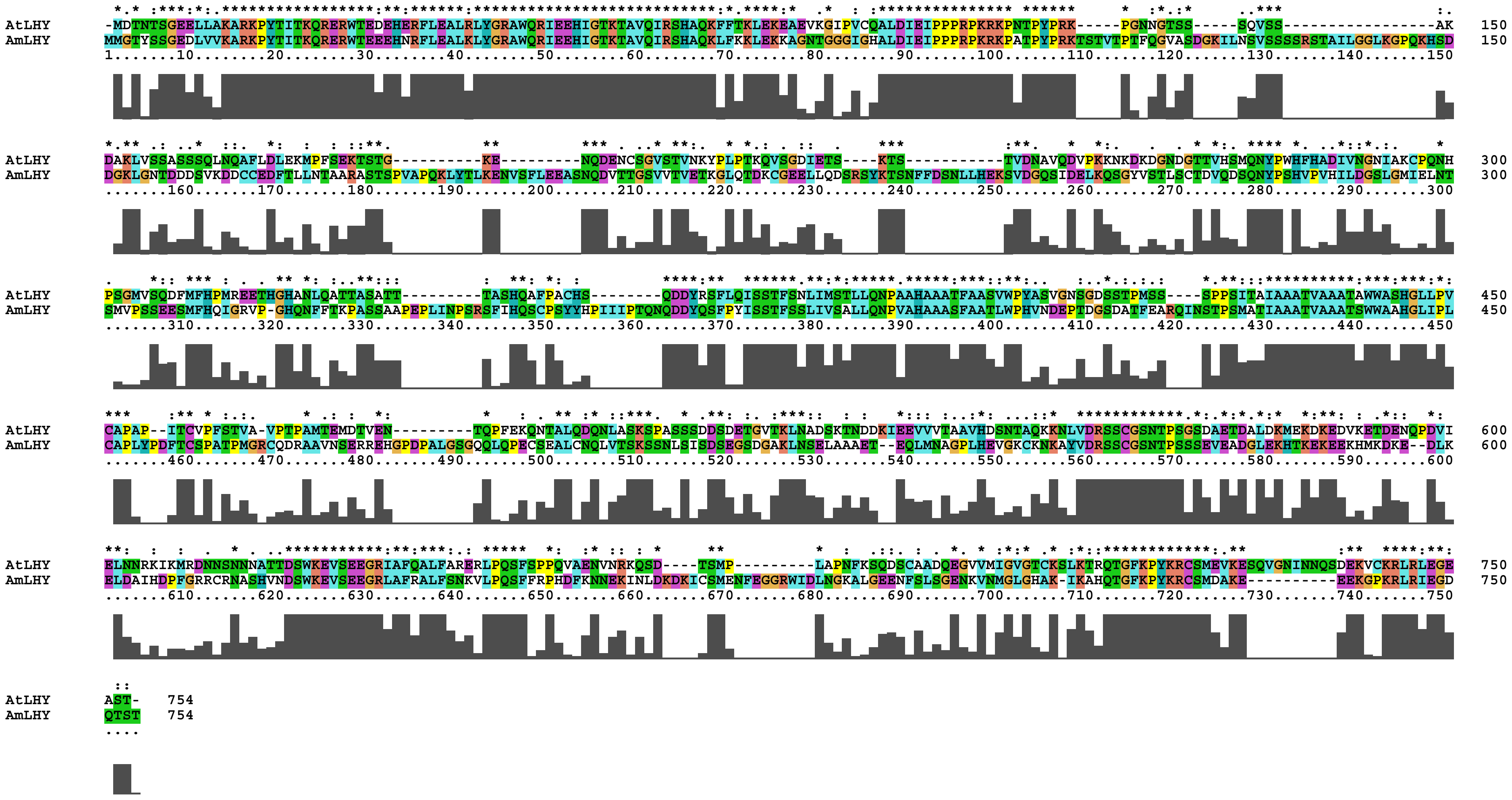

Figure S3

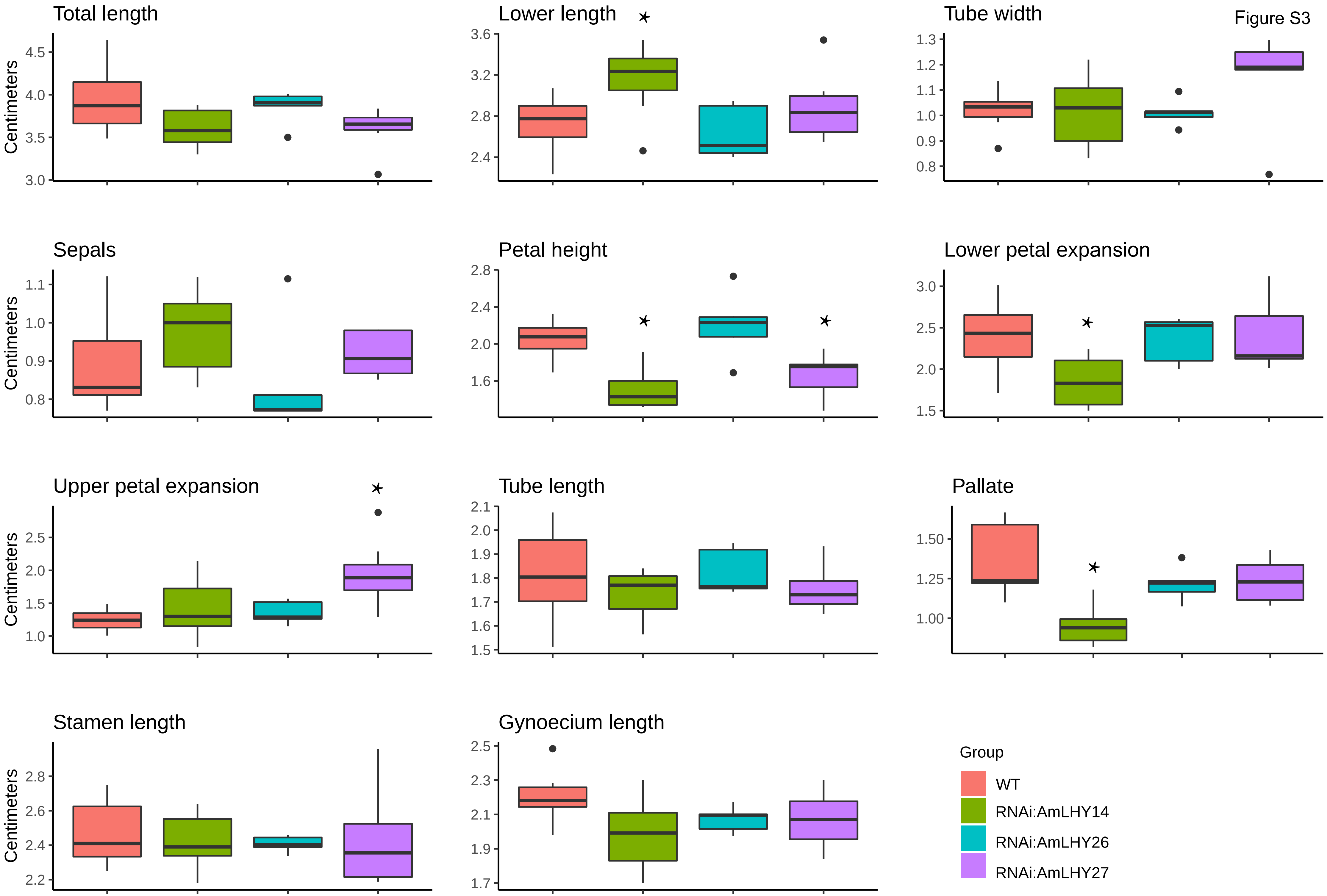
Total length
Lower length
Tube width
Sepals
Petal height
Lower petal expansion
Upper petal expansion
Tube length
Pallate
Stamen length
Gynoecium length
Centimeters
4.5
4.0
3.5
3.0
3.6
3.2
2.8
2.4
1.3
1.2
1.1
1.0
0.9
0.8
1.1
1.0
0.9
0.8
2.8
2.4
2.0
1.6
3.0
2.5
2.0
1.5
2.5
2.0
1.5
1.0
2.1
2.0
1.9
1.8
1.7
1.6
1.5
1.50
1.25
1.00
2.8
2.6
2.4
2.2
2.5
2.3
2.1
1.9
1.7
Group
WT
RNAi:AmLHY14
RNAi:AmLHY26
RNAi:AmLHY27

Figure S4

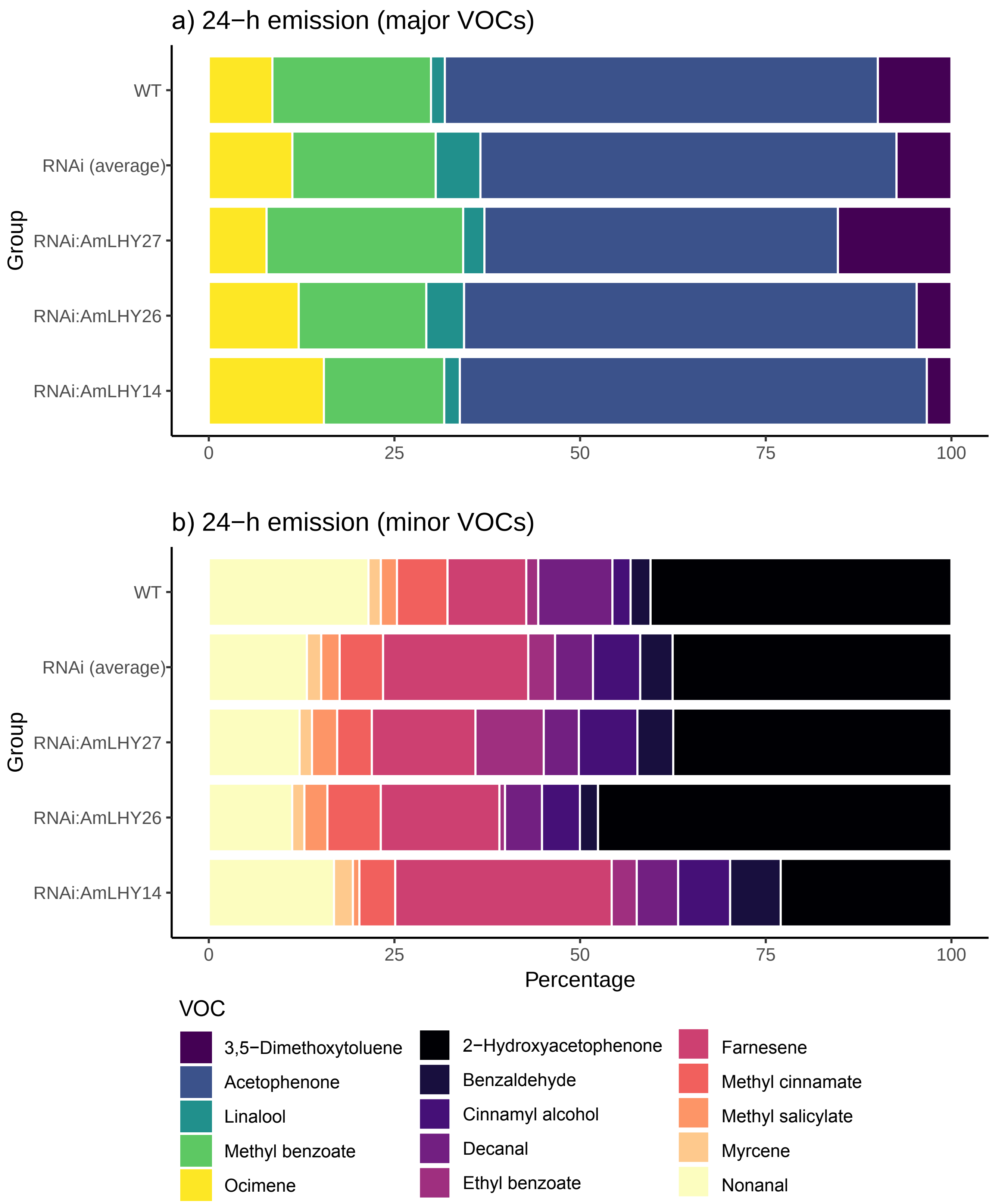
a) 24−h emission (major VOCs)
b) 24−h emission (minor VOCs)
Group
WT
RNAi (average)
RNAi:AmLHY27
RNAi:AmLHY26
RNAi:AmLHY14
0
25
50
75
100
Percentage
VOC
3,5−Dimethoxytoluene
Acetophenone
Linalool
Methyl benzoate
Ocimene
2−Hydroxyacetophenone
Benzaldehyde
Cinnamyl alcohol
Decanal
Ethyl benzoate
Farnesene
Methyl cinnamate
Methyl salicylate
Myrcene
Nonanal

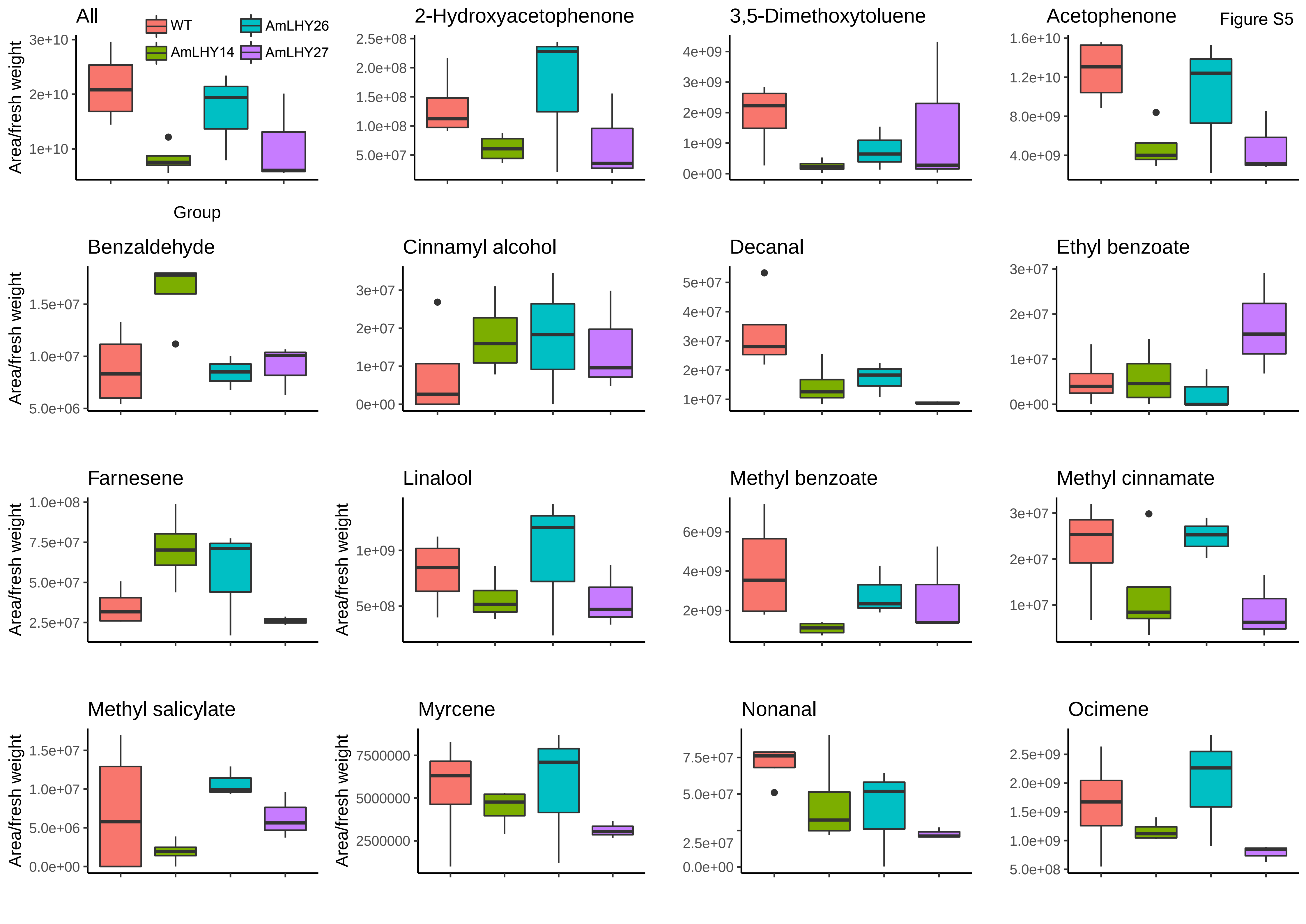
Figure S5
All
WT
AmLHY14
AmLHY26
AmLHY27
Group
Area/fresh weight
2-Hydroxyacetophenone
3,5-Dimethoxytoluene
Acetophenone
Benzaldehyde
Cinnamyl alcohol
Decanal
Ethyl benzoate
Farnesene
Linalool
Methyl benzoate
Methyl cinnamate
Methyl salicylate
Myrcene
Nonanal
Ocimene

Supplementary Table S1. Accession numbers of proteins used in phylogenetic reconstruction.

Specie	Accession	Protein
Arabidopsis thaliana (At)	AT1G01060	LATE ELONGATED HYPOCOTYL (LHY)
	OAP07470.1	CIRCADIAN CLOCK ASSOCIATED 1 (CCA1)
	OAO92169.1	REVEILLE 1 (RVE1)
	OAO90963.1	REVEILLE 2 (RVE2)
	AT1G01520	REVEILLE 3 (RVE3)
	gi|75324470|	REVEILLE 4 (RVE4)
	gi|576017836|	REVEILLE 5 (RVE5)
	AT5G52660	REVEILLE 6 (RVE6)
	gi|576017949|	REVEILLE 7 (RVE7)
	OAP05994.1	REVEILLE 8 (RVE8)
Amborella trichopoda (Amt)	XP_006829218.1	LATE ELONGATED HYPOCOTYL (LHY)
Antirrhinum majus (Am)	Am03g44570	LATE ELONGATED HYPOCOTYL (LHY)
	LT978479.1	
	Am04g07010	REVEILLE 1A-like (RVE1A-like)
	Am06g21890	REVEILLE 1B-like (RVE1B-like)
	Am04g18800	REVEILLE 2A-like (RVE2A-like)
	Am06g21900	REVEILLE 2B-like (RVE2B-like)
	Am02g37040	REVEILLE 3/5A-like (RVE3/5A-like)
	Am03g44150	REVEILLE 3/5B-like (RVE3/5B-like)
	Am08g25850	REVEILLE 4/8-like (RVE4/8-like)
Glycine soja (Gs)	KHN11366.1	LATE ELONGATED HYPOCOTYL (LHY)
Hordeum vulgare (Hv)	AEW48245.1	CIRCADIAN CLOCK ASSOCIATED 1 (CCA1)
Nicotiana attenuata (Na)	OIS99379.1	LATE ELONGATED HYPOCOTYL (LHY)
Nicotiana sylvestris (Ns)	XP_009794791.1	PREDICTED: protein LHY
Oryza sativa (Japonica Group) (Os)	XP_015649847.1	LHY isoform X4
Ostreococcus tauri (Ot)	AAU14271.1	LHY-like protein, partial
Petunia hybrida (Ph)	AKL88450.1	LATE ELONGATED HYPOCOTYL (LHY)
Phaseolus vulgaris (Pv)	CAD12767.2	LATE ELONGATED HYPOCOTYL (LHY)
Physcomitrella patens (Pp)	BAI39991.1	CIRCADIAN CLOCK ASSOCIATED 1A, partial
	BAI39992.1	CIRCADIAN CLOCK ASSOCIATED 1B, partial
Vigna radiata (Vr)	XP_014521593.1	LATE ELONGATED HYPOCOTYL (LHY)
Vigna unguiculata (Vu)	XP_027906072.1	LATE ELONGATED HYPOCOTYL–like (LHY)
Zea mays (Zm)	NP_001131529.2	LATE ELONGATED HYPOCOTYL (LHY)
	ADU60099.1	CIRCADIAN CLOCK ASSOCIATED 1 (CCA1)
	AQK41466.1	CIRCADIAN CLOCK ASSOCIATED2 (CCA2)

Table S2 BLAST analysis. Arabidopsis accessions (Query ID) were used to identify REVEILLE (RVE, Protein column) sequences in the snapdragon genome database (http://bioinfo.sibs.ac.cn/Am/index.php). RVE names were assigned based on a tree generated by simple phylogeny (EMBL-EBI, https://www.ebi.ac.uk/).

Query ID	Protein	Snapdragon accession	P identity	e value	Assigned name
OAO92169.1	AtRVE1	Am04g07010	86.49	$9e^{-23}$	AmRVE1/2/7A
		Am06g21890	47.15	$5e^{-20}$	AmRVE1/2/7B
OAO90963.1	AtRVE2	Am04g18800	80.56	$2e^{-23}$	AmRVE1/2/7C
		Am06g21900	51.00	$2e^{-20}$	AmRVE1/2/7D
AT1G01520	AtRVE3	Am08g25850	76.79	$1e^{-18}$	AmRVE4/8
		Am02g37040	88.37	$4e^{-17}$	AmRVE3/5B
gi|75324470|	AtRVE4	Am04g07010	72.97	$8e^{-14}$	AmRVE1/2/7A
		Am04g18800	70.27	$1e^{-13}$	AmRVE1/2/7C
gi|576017836|	AtRVE5	Am03g44150	76.60	$1e^{-15}$	AmRVE3/5A
		Am02g37040	79.55	$4e^{-15}$	AmRVE3/5B
AT5G52660	AtRVE6	Am04g07010	72.97	$3e^{-14}$	AmRVE1/2/7A
		Am04g18800	70.27	5e-^{14}	AmRVE1/2/7C
gi|576017949|	AtRVE7	Am04g18800	67.57	$2e^{-21}$	AmRVE1/2/7C
		Am06g21890	44.86	$6e^{-20}$	AmRVE1/2/7B
OAP05994.1	AtRVE8	Am04g07010	78.38	$2e^{-15}$	AmRVE1/2/7A
		Am04g18800	72.97	$5e^{-15}$	AmRVE1/2/7C

Supplementary Table S3. PCR primers used in the current study

Gene	Forward (5’ – 3’)	Reverse (5’ – 3’)
RNAi:AmLHY	TCTGGATGAAACAAGTGTCC	TAAAAAGTTTCTGGGCATGA
NPTII	CCTGCTTGCCGAATATCATGGTGG	CGAAATCTCGTGATGGCAGGTTGG
AmUBI	CCGATGGAAGTATATGTTTG	CCAGTCTTACAAGACCTAGAAGCTC
AmLHY	TTACTTGCTCTCCGGCAACT	TTGAAGTCACAAGCTGATTGC

Supplementary Table S4. Analysis of floral parameters in non-transgenic and transgenic lines. Parameters were analyzed using a Student's t test. *P* value < 0.05 indicates a significant difference between non-transgenic and transgenic snapdragon flowers, significance levels are indicated with one asterisks (* for $p < 0.05$), two asterisks (** for $p < 0.001$) and three asterisks (*** for $p < 0.0001$).

Parameter	RNAi:AmLHY14	RNAi:AmLHY26	RNAi:AmLHY27
Total length	0.037*	0.858	0.069
Lower length	0.014*	0.486	0.210
Tube width	0.927	0.927	0.001*
Sepal length	0.337	0.646	0.762
Petal height	0.000***	0.060	0.004*
Lower petal expansion	0.005*	0.885	0.885
Upper petal expansion	0.137	0.419	0.000***
Tube length	0.404	0.881	0.404
Pallate	0.000***	0.258	0.160
Stamen length	0.974	0.974	0.974
Gynoecium length	0.048*	0.278	0.278

Supplementary Table S5. Retention time in minutes (RT), CAS number of detected volatiles.

RT	CAS	Volatile
4.611	100-52-7	Benzaldehyde
5.235	123-35-3	Myrcene
6.296	3779-61-1	Ocimene
7.701	124-19-6	Nonanal
7.844	98-86-2	Acetophenone
8.411	93-58-3	Methyl benzoate
8.511	78-70-6	Linalool
8.778	118-93-4	2-Hydroxyacetophenone
9.387	119-36-8	Methyl salicylate
9.549	112-31-2	Decanal
9.839	93-89-0	Ethyl benzoate
10.554	4179-19-5	3,5-Dimethoxytoluene
11.192	104-54-1	Cinnamyl alcohol
12.349	19713-73-6, 103-26-4	Methyl cinnamate
14.006	502-61-4	Farnesene

Supplementary Table S6. Analysis of daily emitted volatiles with the algorithm JTK_CYCLE in wild type (WT) and RNAi lines. Phase indicates the time point (ZT hours) with maximum emission. Shift indicates if the maximum emission of a VOC was advanced (A), delayed (D) or did not changed (NC) compared to the wild type. No data indicates that a parameter could not be determined.

	WT	RNAi:AmLHY14		RNAi:AmLHY26		RNAi:AmLHY27	
Volatile	Phase	Phase	Shift	Phase	Shift	Phase	Shift
Myrcene	0	6	D	6	D	6	D
Decanal	3	3	NC	3	NC	3	NC
Ocimene	3	6	D	6	D	6	D
2-Hydroxyacetophenone	6	6	NC	6	NC	6	NC
3,5-Dimethoxytoluene	6	6	NC	6	NC	6	NC
Linalool	6	6	NC	6	NC	6	NC
Nonanal	6	6	NC	6	NC	6	NC
Methyl cinnamate	6	3	A	6	NC	3	A
Cinnamyl alcohol	9	9	NC	9	NC	9	NC
Farnesene	9	6	A	6	A	6	A
Methyl benzoate	15	18	D	18	D	18	D
Acetophenone	15	12	A	12	A	12	A
Methyl salicylate	15	21	D	21	D	21	D
Benzaldehyde	18	12	A	12	A	12	A
Ethyl benzoate	No data	No data		No data		No data	
